# Berberine Regulated Lipid Metabolism in the Presence of C75, Compound C, and TOFA in Breast Cancer Cell Line MCF-7

**DOI:** 10.1155/2015/396035

**Published:** 2015-08-13

**Authors:** Wen Tan, Zhangfeng Zhong, Shengpeng Wang, Zhanwei Suo, Xian Yang, Xiaodong Hu, Yitao Wang

**Affiliations:** ^1^School of Pharmacy, Lanzhou University, 199 West Donggang Road, Lanzhou, Gansu 730000, China; ^2^Institute of Chinese Medical Sciences, State Key Laboratory of Quality Research in Chinese Medicine, University of Macau, Macau

## Abstract

Berberine interfering with cancer reprogramming metabolism was confirmed in our previous study. Lipid metabolism and mitochondrial function were also the core parts in reprogramming metabolism. In the presence of some energy-related inhibitors, including C75, compound C, and TOFA, the discrete roles of berberine in lipid metabolism and mitochondrial function were elucidated. An altered lipid metabolism induced by berberine was observed under the inhibition of FASN, AMPK, and ACC in breast cancer cell MCF-7. And the reversion of berberine-induced lipid suppression indicated that ACC inhibition might be involved in that process instead of FASN inhibition. A robust apoptosis induced by berberine even under the inhibition of AMPK and lipid synthesis was also indicated. Finally, mitochondrial function regulation under the inhibition of AMPK and ACC might be in an ACL-independent manner. Undoubtedly, the detailed mechanisms of berberine interfering with lipid metabolism and mitochondrial function combined with energy-related inhibitors need further investigation, including the potential compensatory mechanisms for ATP production and the upregulation of ACL.

## 1. Introduction

Lipid metabolism plays discrete roles in cancer reprogramming metabolism, including the conventional roles, namely, membrane structure generation, providing signaling molecules and posttranslational modification of proteins, and the novel roles, namely, participating in autophagy and metastasis, involved in microenvironment and angiogenesis. It was systematically expounded in Baenke's review [1]. Even lipolysis also contributes to cancer pathogenesis [2]. Collectively, lipid metabolism in cancer cells was context dependent and would generate intricate connections in cancer reprogramming metabolism. Mitochondrial function was another core part of cancer metabolism [3–6] and was also implied in many aspects, including therapeutic resistance [7–17], autophagy [18–21], drug sensitivity [22–24], apoptosis [25–38], metastasis [39–42], and angiogenesis [43]. Mitochondrial function was regarded as a metabolic symbiosis between tumor stromal cells and epithelial cancer cells in human breast cancer [5]. The final exhibited mitochondrial function might be a result of concerted action of the dysfunction of stromal cells and the biogenesis of epithelial cancer cells. Our study focused on the effect of berberine on lipid metabolism and mitochondrial function in presence of C75, compound C, and TOFA in cancer cells, which is relatively rare. We tested the induced apoptosis, lipid droplets content, and mitochondrial function alteration in presence of these inhibitors, in order to explore the underlying mechanisms of berberine regulating reprogramming metabolism of cancer cells when combined with conventional inhibitors.

C75 (4-methylene-2-octyl-5-oxotetra-hydrofuran-3-carboxylic acid) is an analogue of cerulenin [44] and is a stable inhibitor of fatty acid synthase (FASN). In pancreatic ductal adenocarcinoma cells Colo357, C75 induced increased apoptosis in a dose-dependent manner* in vitro* and 50% of reduction of primary pancreatic tumor weight in mouse orthotopic tumor* in vivo*, accompanied by an antimetastasis effect [45]; similar results of growth suppression were also observed in LNCaP prostate cancer cell* in vitro* and in LNCaP/tk-luc bearing animal model* in vivo* [46]. And, in breast cancer cell lines, C75 inhibited FASN and stimulated beta-oxidation through carnitine palmitoyltransferase-1 (CPT-1) regulation [47, 48] or directly suppressing HER2 and FASN phosphorylation [49]. And this similar efficacy was also observed in lung cancer cells A549 [50]. Compound C (also called dorsomorphin, 6-[4-(2-piperidin-1-yl-ethoxy)-phenyl]-3-pyridin-4-yl-pyrazolo [1,5-a] pyrimidine) is a reversible ATP-competitive inhibitor of adenosine 5′-monophosphate- (AMP-) activated protein kinase (AMPK) [51]. It induced apoptosis in A549, SMMC-7721, and HeLa cells and exerted transcriptional repression through increasing p53 expression and decreasing the expression of Bcl-2 and Bcl-xl and the phosphorylation of eIF2*α* on Ser51 [52]. Compound C also induced autophagy in cancer cells, including human glioma cells U251, rat glioma cells C6, mouse fibrosarcoma L929, and mouse melanoma cells B16. Notably, the routine AMPK-dependent manner was challenged and discussed in some studies. An induced protective autophagy through AMPK inhibition-independent blockade of Akt/mTOR pathway was presented [51]. And this AMPK-independent manner was also illustrated in the induced necroptosis and autophagy of glioma cells [53], in the sensitization of TRAIL-induced apoptosis in Caki human renal cancer cells [54], and in the inhibition of transcriptional activation of UPR-targeted genes [55]. And in human colorectal cancer cells, including HCT116, DLD-1, SW480, and KM12C, an induced apoptotic or autophagic death was observed simultaneously [56]. The concurrent effect of apoptosis and autophagy by compound C was also found in skin cancer cells and was demonstrated by means of p53 status [57]. TOFA (5-tetradecyloxy-2-furoic acid) is an allosteric inhibitor of acetyl-CoA carboxylase (ACC), also reducing endogenous fatty acid for phospholipid composition of cell membrane [58]. Initially, TOFA showed an inhibition on fatty acid synthesis in hepatocytes, rat liver homogenates, or male rat liver [59–62], and now it showed a wider range of proliferation inhibition on cancer cells, including ovarian cancer cells COC1 and COC1/DDP [63], human prostatic carcinoma cells LNCaP [58], lung cancer cells NCI-H460, and colon carcinoma cells HCT-8 and HCT-15 [64].

Referring to the interactions between berberine and C75, compound C and TOFA in cancer cells, a definitive conclusion warranted more caution because of the insufficient researches. In liver cells HL-7702, the induced Raf-1 signaling stimulation by berberine was blocked by compound C [26]. And in human hepatoma cell line HepG2, compound C suppressed apoptosis and autophagy induced by berberine [65] but showed no influence on the stimulated glucose utilization and lactate production induced by berberine [66]. Our study emphasized the regulation of berberine on lipid metabolism and mitochondrial function in presence of energy-related inhibitors in breast cancer cells MCF-7, in order to elucidate the underlying mechanisms of berberine interfering with cancer reprogramming metabolism.

## 2. Materials and Methods

### 2.1. Cell Culture and Reagents

Human breast cancer cell line MCF-7 was purchased from American Type Culture Collection (America) and was cultured with RPMI1640 medium containing fetal bovine serum (10%), penicillin (100 units/mL), and streptomycin (100 *μ*g/mL), at 37°C in a humidified atmosphere of 5% CO_2_ in air.

### 2.2. Cell Viability Assay

MTT assay was conducted to determine cell growth and viability. MCF-7 cells were seeded in 96-well plate at density of 1 × 10^5^/mL. After cell adhesion, a 24-hour serum starvation was carried out for synchronization before drug treatment. Pretreatments of C75, CC, and TOFA for 3 hours were conducted; the concentrations of these stocks were 5 mg/mL, 4 mg/mL, and 5 mg/mL, respectively, which were soluble in DMSO. And the dilutions were 1 : 1000, 1 : 1000, and 1 : 500, respectively, for C75, CC, and TOFA. Berberine treatment duration was 24 hours, and concentrations were 10 *μ*M or 25 *μ*M as required. Cell viability was determined under 570 nm and expressed as percentage of control.

### 2.3. Mitochondrial Membrane Potential Assay

MCF-7 cells were seeded in 96-well plate at density of 1 × 10^5^/mL. After cell adhesion, a 24-hour serum starvation was carried out for synchronization. Drug treatment was according to the mentioned above. Cells were incubated with JC-1 probe (working concentration is 2.5 *μ*g/mL) at 37°C for 1 hour. Determination was conducted under Ex/Em 550 nm/600 nm for red fluorescence and Ex/Em 485 nm/535 nm for green fluorescence, and the ratio of red and green was calculated.

### 2.4. ROS Level Assay

MCF-7 cells were seeded in 96-well plate at density of 1 × 10^5^/mL. After cell adhesion, drug treatment was according to the mentioned above. Cells were incubated with H_2_DCF-DA probe (stock concentration is 1 mM and working concentration is 10 *μ*M) for 1 hour and determined by multifunctional microplate reader SpectraMax M5 under Ex/Em 485 nm/530 nm. Then the diluted Hoechst 33342 was determined by M5 under Ex/Em 355 nm/465 nm.

### 2.5. Lipid Droplets Assay

MCF-7 cells were seeded in 96-well plate at density of 1 × 10^5^/mL. After cell adhesion, drug treatment was according to the mentioned above. Cells were fixed with formalin (10%) and stained in Oil-Red-O (sigma, O0625) solution (stock solution is 5 g/L, dissolved in 2-isopropanol; ratio of the working solution is 6 : 4 [Oil-Red-O stock: MilliQ]) for 1 hour. Cells were rinsed with 60% propanol briefly, then with dH_2_O exhaustively. Isopropanol was added to determine lipid droplets content in cells. The extracted dye was transferred into a new 96-well plate for determination under 490 nm by M5, and the values were presented as the percentage of control.

### 2.6. Mitochondrial Function Assay

MCF-7 cells were seeded in 96-well plate at density of 1 × 10^5^/mL. After cell adhesion, a 24-hour serum starvation was carried out for synchronization. Drug treatment was according to the mentioned above. Mitochondrial deep red probe solution was incubated and protected from light for 1 hour (working concentration is 1 *μ*M in PBS). Then, cells were fixed with paraformaldehyde (3.7%) for 45 mins and were incubated with triton X-100 (0.2%) under room temperature for 30 mins. Function determination was conducted under Ex/Em 644 nm/665 nm with multifunctional microplate reader SpectraMax M5 and DAPI staining determination under Ex/Em 355 nm/460 nm.

### 2.7. ATP Production Assay

MCF-7 cells were seeded in Petri dish at density of 2 × 10^6^/mL. After cell adhesion, drug treatment was according to the mentioned above. According to the protocol of ATP colorimetric assay kit, determination was conducted under absorbance wavelength of 570 nm and values were presented as the percentage of control.

### 2.8. Western Blotting

MCF-7 cells were seeded in 45 mm flask at density of 1 × 10^6^/mL. After cell adhesion, drug treatment was according to the mentioned above. Cell lysate was collected and spun for 5 mins under 4°C, and the total protein content was determined according to BCA kit protocol. The equal weight of total protein was subjected to SDS-PAGE (6%) gels and transferred to methanol-treated PVDF membrane. One-hour blocking in nonfat milk (5%), 2-hour incubation for the first antibodies (cell signaling, 1 : 1000 dilution), and 1-hour incubation for the secondary antibodies were performed. ECL advanced western blotting detection kit (Amersham) was used for visualization, and the band density was normalized by the density of beta-actin.

## 3. Results and Discussion

### 3.1. Results

#### 3.1.1. Cell Viability of Human Breast Cancer Cells MCF-7 Was Not Inhibited by Pretreatments of C75, Compound C, and TOFA, Even in the Presence of Berberine

**Figure 1:**
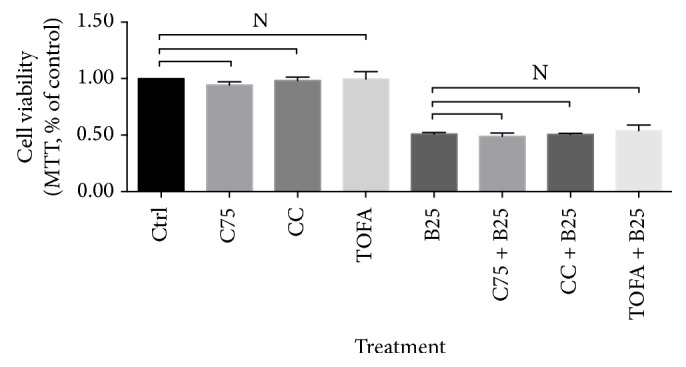
Cell viability of human breast cancer cell line MCF-7 in presence of C75, CC, and TOFA. Cell viability was obtained by MTT assay after pretreatment of C75 (5 *μ*g/mL), CC (4 *μ*g/mL), and TOFA (10 *μ*g/mL) for 3 hours and treatment of berberine (25 *μ*M) for 24 hours. Cell viability was presented as percentage of control, and significance difference was calculated by Student's test, *p* value ^*∗*^ < 0.05.

FASN inhibitor C75, AMPK inhibitor compound C (CC), and ACC inhibitor TOFA were conducted to test cell viability influence in human breast cancer cell line MCF-7. After 3-hour pretreatments of C75, CC, and TOFA, 24-hour cell viability was determined by MTT assay with no significance. And, in the presence of berberine (25 *μ*M for 24 hours), these pretreatments also showed no significant variance, which was shown in Figure 1.

#### 3.1.2. Berberine Decreased the Elevated Mitochondrial Membrane Potential Induced by Compound C or TOFA

**Figure 2:**
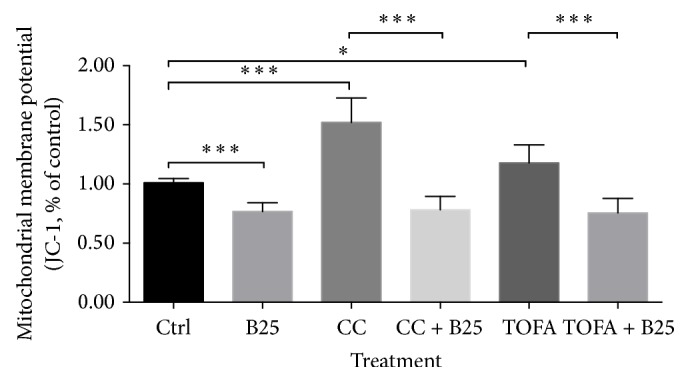
Mitochondrial membrane potential alteration of MCF-7 cells in presence of CC and TOFA. Mitochondrial membrane potential of MCF-7 cells was assayed by JC-1 probe after pretreatment of CC (4 *μ*g/mL) and TOFA (10 *μ*g/mL) for 3 hours and treatment of berberine (25 *μ*M) for 24 hours. Mitochondrial membrane potential alterations were presented as percentage of control, and all significant difference was calculated by Student's test and presented as ^*∗*^ and ^*∗∗∗*^, *p* value <0.05 and <0.005.

Berberine significantly decreased mitochondrial membrane potential in human breast cancer cells MCF-7. The pretreatments of CC and TOFA dramatically increased mitochondrial membrane potential. But the subsequent treatment of berberine at dosage of 25 *μ*M for 24 hours reversed this increase with significance, as shown in Figure 2.

#### 3.1.3. Berberine Interfered with ROS Generation after CC or TOFA Pretreatment

**Figure 3:**
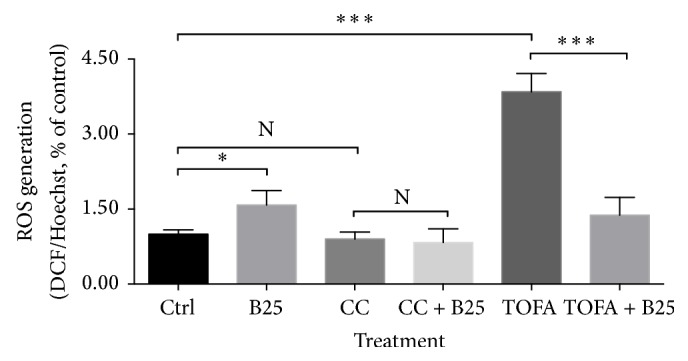
ROS level of MCF-7 cells in presence of CC and TOFA. ROS level of MCF-7 cells was assayed by H_2_DCF-DA/Hoechst 33342 after pretreatment of CC (4 *μ*g/mL) and TOFA (10 *μ*g/mL) for 3 hours and treatment of berberine (25 *μ*M) for 24 hours. ROS generation was presented as percentage of control, and all significant difference was calculated by Student's test and presented as ^*∗*^ and ^*∗∗∗*^, *p* value <0.05 and <0.005.

Although berberine increased ROS level in MCF-7 cells, it remarkably reduced ROS level induced by TOFA, as shown in Figure 3. While in CC pretreatment groups, ROS content was not disturbed by CC pretreatment and also not disturbed by subsequent treatment of berberine at dosage of 25 *μ*M for 24 hours.

#### 3.1.4. TOFA and C75 Influenced the Efficacy of Berberine on Lipid Droplets

**Figure 4:**
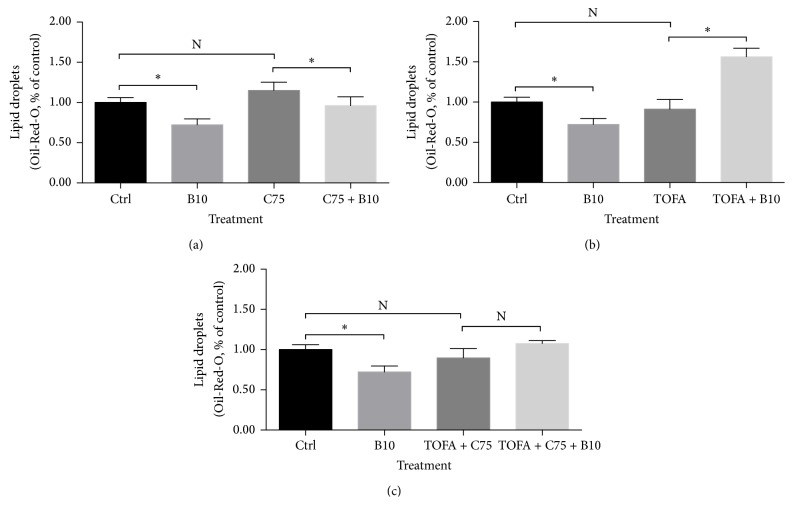
Lipid droplets alteration of MCF-7 cells in presence of C75, TOFA, and the combination of both. Lipid droplets alteration of MCF-7 cells was assayed by Oil-Red-O staining after pretreatment of C75 (5 *μ*g/mL) (a), TOFA (10 *μ*g/mL) (b), and the combination of both (c) for 3 hours and treatment of berberine (10 *μ*M) for 24 hours. Lipid droplets content was presented as percentage of control, and all significant difference was calculated by Student's test and presented as ^*∗*^, *p* value <0.05.

No significant variations were found in lipid droplets content after the treatment of C75, TOFA, and the combination of both in MCF-7 cells, as shown in Figures 4(a), 4(b), and 4(c). Berberine treatment inhibited the content of lipid droplets significantly in MCF-7 cells, and the less lipid droplets were also observed in MDA-MB-231 cells in our previous study. But, in the presence of C75, TOFA, or the combination of both, the decreased content of lipid droplets induced by berberine changed. Briefly, berberine increased the content of lipid droplets after TOFA pretreatment and decreased the content of lipid droplets after C75 pretreatment. And, after the pretreatment of the combination of C75 and TOFA, berberine showed no significance on lipid droplets alteration.

#### 3.1.5. In the Presence of C75, CC, and TOFA, the Berberine-Induced Mitochondrial Function Alterations Changed

**Figure 5:**
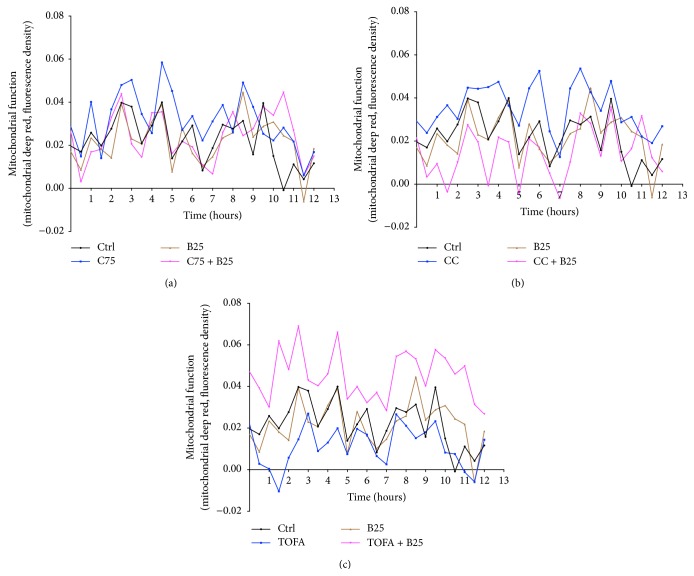
Mitochondrial function alteration of MCF-7 cells in presence of C75, CC, and TOFA. Mitochondrial function alteration of MCF-7 cells was assayed by mitochondrial deep red real-time determination after pretreatment of C75 (5 *μ*g/mL) (a), CC (4 *μ*g/mL), (b) and TOFA (10 *μ*g/mL) (c) for 3 hours and treatment of berberine (25 *μ*M) for 12 hours. During 12-hour duration, a real-time alteration of mitochondrial function was detected and the curves were presented as percentage of responding value on the initial time point.

In the absence of C75, CC, and TOFA, the berberine-induced mitochondrial function alterations changed in MCF-7 cells using a real-time determination, as shown in Figure 5. However, in the presence of C75, mitochondrial function elevated moderately and the subsequent berberine treatment suppressed this elevation slightly in Figure 5(a). And, in the presence of CC, the elevated mitochondrial function was suppressed dramatically by berberine treatment in Figure 5(b). Conversely, in the presence of TOFA, mitochondrial function was inhibited remarkably by pretreatment and the subsequent berberine treatment reversed this inhibition substantially in Figure 5(c).

#### 3.1.6. CC and TOFA Influenced the Efficacy of Berberine on ATP Production

**Figure 6:**
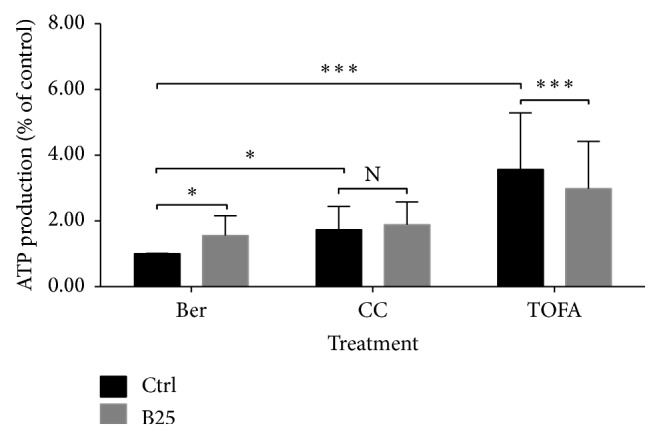
ATP production of MCF-7 cells in presence of CC and TOFA. ATP production of MCF-7 cells was assayed by ATP assay kit after pretreatment of CC (4 *μ*g/mL) and TOFA (10 *μ*g/mL) for 3 hours and treatment of berberine (25 *μ*M) for 24 hours. ATP production was presented as percentage of control, and all significant difference was calculated by Student's test and presented as ^*∗*^ and ^*∗∗∗*^, *p* value <0.05 and <0.005.

Berberine significantly increased ATP level in MCF-7 cells in our previous study. And CC or TOFA pretreatment could also increase ATP level in MCF-7 cells. But, in the presence of CC, this elevation induced by berberine treatment was not observed as shown in Figure 6. And, in the presence of TOFA, this elevation was reversed significantly by berberine treatment.

#### 3.1.7. Berberine Increased Phosphor ACL despite the Presence of CC or TOFA

**Figure 7:**
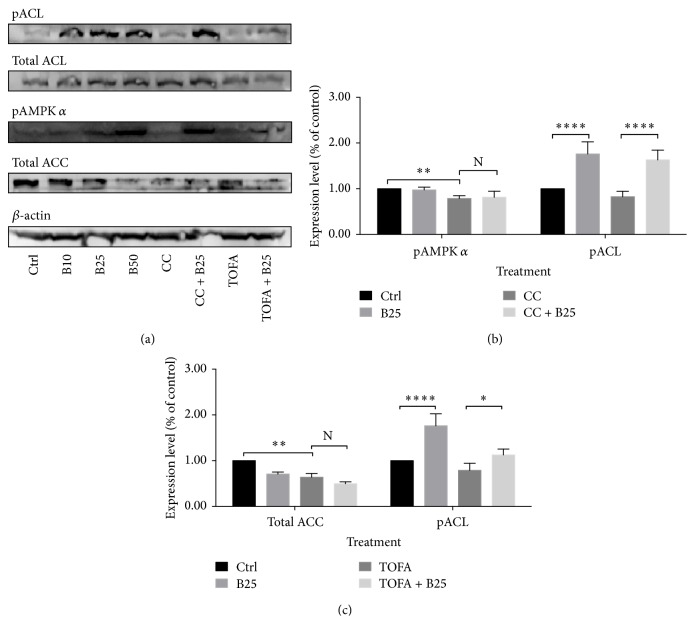
Phosphor ACL alteration of MCF-7 cells in presence of CC and TOFA. Phosphor ACL alteration of MCF-7 cells was assayed by western blotting after pretreatment of CC (4 *μ*g/mL) and TOFA (10 *μ*g/mL) for 3 hours and treatment of berberine (25 *μ*M) for 24 hours. The expression alterations of the corresponding proteins were visualized by band densitometry (a) and were presented as percentage of control (b). All significant difference was calculated by Student's test and presented as ^*∗*^, ^*∗∗*^, and ^*∗∗∗∗*^, *p* value <0.05, <0.01, and <0.001.

Berberine increased phosphor ACL in MCF-7 cells. Even after CC inhibited phosphor AMPK significantly, the elevated expression of phosphor ACL induced by berberine was still observed in Figure 7(b). Similar situation was also observed in TOFA pretreatment. After TOFA inhibited total ACC significantly, berberine treatment increased the expression of phosphor ACL, the same as the increase in the absence of TOFA in Figure 7(c).

### 3.2. Discussion

#### 3.2.1. Berberine Induced Apoptosis after C75, CC, and TOFA Pretreatments

No significances were observed in cell viability after pretreatments of C75, CC, and TOFA despite the absence or presence of berberine. The MTT assay for cell viability excluded the potential impact of inhibitors on cell growth, which facilitated the unilateral mechanism study of berberine on cell lipid metabolism. In the absence of CC or TOFA, berberine induced a significant reduction of mitochondrial membrane potential in MCF-7 cells. However, in the presence of CC or TOFA, this substantial reduction induced by berberine was still observed with significance, even with more significant differences. In other words, the suppression induced by berberine still lingered in independent pattern with the existence of CC or TOFA. Given that CC is an inhibitor of AMPK and TOFA is an inhibitor of ACC, the decreased mitochondrial membrane potential independent of CC or TOFA indicated a slight relationship between mitochondrial depolarization and AMPK inhibition or ACC inhibition. Actually, AMPK regulation or ACC inhibition by berberine was also found in others' studies [67–69]. And, in our study, this independent manner indicated that berberine efficacy on mitochondrial membrane potential was not associated with AMPK or ACC inhibition. Mitochondrial membrane potential assay by JC-1 probe also determined the proportion of nonapoptosis cells. This robust suppression of berberine indicated the induced apoptosis even under AMPK inhibition and lipid synthesis inhibition. ROS generation was another hallmark event of apoptosis, and ROS level also elevated after berberine treatment. But, in the presence of CC, the elevation induced by berberine was abolished, which indicated a blockage of berberine-induced apoptosis. However, in the presence of TOFA, the elevation induced by berberine was reversed significantly. The substantial increase of ROS level induced by TOFA was inhibited dramatically by berberine treatment. The efficacy of stimulating ROS level by berberine in the absence of TOFA changed into suppressing ROS level in the presence of TOFA. This might suggest a palliation of berberine on TOFA-induced ROS generation, also indicating that berberine might attenuate the TOFA-induced apoptosis.

#### 3.2.2. Berberine Regulated Lipid Metabolism in the Presence of C75 and TOFA

Lipid metabolism was found closely related to membranes synthesis, energy homeostasis, and signaling function for cancer cells [70, 71]. Lipid droplets content was also higher than that of normal tissue. In colorectal cancer, the high level of lipid droplets was regarded as a distinctive mark of cancer stem cells [72]. We chose breast cancer cells MDA-MB-231 to conduct Oil-Red-O staining, because it contained much more lipid droplets and a clearer observation could be obtained. Less lipid droplets were clearly visible after berberine treatment in MDA-MB-231 cells (data were not shown). Then, the lipid droplets content in MCF-7 cells assayed by spectrometer M5 also confirmed the reduced quantity induced by berberine. Despite ACC inhibition by TOFA or FASN inhibition by C75, the lipid droplets content in MCF-7 cells showed no significant alteration. This indicated that the ACC inhibition or FASN inhibition would not directly alter the ultimate lipid droplets content. But, in the presence of TOFA or C75, we found that TOFA reversed the efficacy of berberine on lipid metabolism but not C75. And in the combination treatment of TOFA and C75, because of the existence of TOFA, the abolished efficacy was obtained, as shown in Figure 4 with no significance. Collectively, ACC inhibition by TOFA reversed the suppression of berberine on lipid metabolism, while FASN inhibition was not powerful as ACC inhibition. Actually, in our previous study, berberine was also found to inhibit ACC expression in a dose and time dependent manner in MCF-7 cells [73]. Since berberine and TOFA both inhibited ACC expression, the content of lipid droplets in presence of TOFA needed further investigations.

#### 3.2.3. Berberine Regulated Mitochondrial Function in the Presence of C75, CC, and TOFA

Under the different backgrounds, the efficacy of berberine on mitochondrial function in real time showed different characteristics. Briefly, in the presence of C75 and CC, berberine exhibited suppression on mitochondrial function in varying levels. The former was slight and the latter was conspicuous, but both were suppression. On the contrary, in the presence of TOFA, berberine exhibited stimulation on mitochondrial function with a substantial level. And, in our previous study, the efficacy of berberine on mitochondrial function was prone to be stimulation in a short-term manner [73]. In other words, after AMPK inhibition, the efficacy of berberine transformed into slight suppression on mitochondrial function; after FASN inhibition, the efficacy transformed into suppression with a greater degree; and, after ACC inhibition, the stimulation by berberine was amplified and observed significantly. Therefore, the efficacy of berberine on mitochondrial function was closely related to ACC inhibition and could be blocked by AMPK or FASN inhibition. Since berberine was found to inhibit ACC through phosphorylation in our previous study, TOFA took over the ACC inhibition and amplified stimulation of berberine substantially. In ATP level assay, CC abolished the elevated ATP content induced by berberine and TOFA reversed the elevated ATP significantly. Collectively, the slight suppression on mitochondrial function by berberine in presence of CC was companied with the abolishment of the elevated ATP content; and the remarkable stimulation on mitochondrial function by berberine in presence of TOFA was companied with the reversal of the elevated ATP content. Therefore, the latter, the remarkable stimulation of mitochondrial function accompanied by the reduced ATP content, should deserve more attention than the former. This might be due to the fact that mitochondrial function determination was a real-time assay while ATP level was an end-point assay. ATP level indicated the final quantity in this process compared with the control group, while function assay showed a dynamic change of this process. Although the dynamic process was stimulated, the net output of this process might be reduced finally. In this process, many other compensatory mechanisms might be involved and lead to the final reduction of ATP content in MCF-7 cells. Besides, the upregulated ACL was regarded as a key player in lipid metabolism in cancer cells [74–76], but the berberine-induced ACL upregulation still needed further investigation. The robust upregulation of ACL induced by berberine in the absence or presence of CC and TOFA was observed with significance. TOFA pretreatment blocked the conversion of citrate to malonyl-CoA and the conversion of malonyl-CoA to acetyl-CoA, both of which were catalyzed by ACC. Citrate and acetyl-CoA had no other way out but the acetyl-CoA producing pathway. Finally, the upregulated ACL by berberine leads to an accumulation of acetyl-CoA in cytosol. The CC-induced AMPK inhibition also did not regulate this acetyl-CoA accumulation process.

## 4. Conclusion

Berberine regulated lipid metabolism under the inhibitions of AMPK, FASN, and TOFA in breast cancer cells MCF-7. The robust suppression of mitochondrial membrane potential still lingered in independent pattern with the existence of CC or TOFA, indicating a berberine-induced apoptosis even under AMPK inhibition and lipid synthesis inhibition. A palliation of berberine on TOFA-induced ROS increase also suggested the attenuation on the TOFA-induced apoptosis. Meanwhile, ACC inhibition by TOFA reversed the berberine-induced suppression on lipid metabolism, while FASN inhibition was not as powerful as ACC inhibition on reversing the suppressed lipid metabolism. Besides, the altered mitochondrial function in the presence of CC and TOFA and the robust upregulation of ACL in the absence or presence of CC and TOFA with significance also suggested the interference of berberine with cancer reprogramming metabolism. Undoubtedly, the detailed mechanisms of berberine interfering with lipid metabolism under the condition of energy-related inhibitors needed further investigation, including the potential compensatory mechanisms for ATP production and the upregulation of ACL.
